# Roles of Insulin-Like Growth Factor-1 in Muscle Wasting and Osteopenia in Mice with Hyponatremia

**DOI:** 10.1007/s00223-025-01369-7

**Published:** 2025-04-14

**Authors:** Naoyuki Kawao, Akihito Nishikawa, Daichi Matsumura, Ayaka Yamada, Takashi Ohira, Yuya Mizukami, Hiroshi Kaji

**Affiliations:** 1 Department of Physiology and Regenerative Medicine, Faculty of Medicine, Kindai University, 377-2 Ohnohigashi, Osakasayama, Osaka 589-8511 Japan; 2 Department of Orthopaedic Surgery, Faculty of Medicine, Kindai University, Osakasayama, Japan

**Keywords:** Hyponatremia, IGF-1, Bone loss, Muscle wasting

## Abstract

Hyponatremia is associated with sarcopenia and osteoporosis in elderly individuals. Skeletal muscle releases myokines, which affect distant organs, including bone. However, the detailed mechanisms by which hyponatremia influences muscle and bone remain unclear. We herein investigated the effects of hyponatremia on muscle, bone, and myokines linking muscle to bone in mice treated with 1-desamino-8-D-arginine vasopressin (dDAVP) or furosemide, which induce hyponatremia. Muscle mass and bone mineral density (BMD) were analyzed 8 weeks after the administration of dDAVP or furosemide. dDAVP significantly reduced grip strength, but did not affect tissue weights of gastrocnemius or soleus muscles of mice. Furosemide significantly decreased muscle mass, tissue weights of gastrocnemius and soleus muscles, and grip strength in mice. dDAVP and furosemide decreased trabecular BMD, trabecular bone volume, and cortical BMD at the femurs. Among myokines linking muscle to bone, hyponatremia reduced expression of insulin-like growth factor (IGF)-1 in gastrocnemius and soleus muscles and serum IGF-1 levels in mice. In simple regression analyses, serum IGF-1 levels were positively related to muscle IGF-1 expression, trabecular bone volume, and cortical BMD in mice. The administration of sodium chloride solution to mice ameliorated the decreases in grip strength, muscle mass, trabecular bone volume, cortical BMD, and the levels of muscle and circulating IGF-1 in furosemide-treated mice. The present study demonstrated that hyponatremia induces muscle and bone loss as well as a decrease in muscle IGF-1 expression in mice. The present findings suggest that IGF-1 might be related to muscle wasting and bone loss induced by hyponatremia in mice.

**Supplementary Information:**

The online version contains supplementary material available at 10.1007/s00223-025-01369-7.

## Introduction

Hyponatremia is a commonly encountered electrolyte disorder in clinical practice, and its morbidity is 15–30% in hospitalized patients [1]. Chronic hyponatremia is caused by dehydration, malnutrition, cachexia, chronic diseases, and medication, such as diuretics. A decrease in extracellular body fluid is frequently observed in patients with chronic hyponatremia. Syndrome of inappropriate antidiuretic hormone secretion (SIADH) causes hyponatremia via excessive vasopressin, with an increase in extracellular body fluid due to elevated water reabsorption in the renal collecting duct. Although hyponatremia is often asymptomatic, it is a significant public health problem due to increased adverse outcomes, including mortality, in patients with underlying diseases. In an animal study, hyponatremia reduced bone mineral density (BMD) of femurs by increasing the number of osteoclasts in rats [2]. As its mechanism, an in vitro study revealed that low sodium concentrations directly activate osteoclast formation and bone resorption in mouse cells [3]. A recent study showed that hyponatremia enhances fibroblast growth factor-23 expression in mouse osteoblastic cells [4]. Previous numerous clinical evidence has suggested that hyponatremia is related to osteoporosis and increases risk of falls and fractures in elderly individuals [5–9]. However, the detailed mechanisms of hyponatremia-induced osteoporosis remain unclear.

As for the relationships between hyponatremia and skeletal muscle, hyponatremia reduces muscle mass in aged rats [10]. A previous clinical study revealed that mild hyponatremia is related to low muscle mass and impaired physical function in elderly individuals [11]. Markaki et al. reported that decreased handgrip strength was associated with decreased predialysis serum sodium levels in renal failure patients on hemodialysis [12]. These studies suggest that even mild hyponatremia causes muscle wasting. However, the mechanisms by which hyponatremia affects muscle metabolism and function have not yet been elucidated in detail.

Accumulating evidence suggests that sarcopenia is highly comorbid with osteoporosis in elderly individuals [13]. Based on the clinical studies regarding sarcopenia and osteoporosis, crosstalk between muscle and bone has been studied. Skeletal muscle releases humoral myokines, such as insulin-like growth factor (IGF)-1, fibroblast growth factor (FGF)-2, irisin, myostatin, follistatin, transforming growth factor (TGF)-β, interleukin (IL)-6, and activin A [13]. These myokines affect local and distant organs, including bone, via the circulation. We previously reported that follistatin, olfactomedin 1, and Dickkopf-related protein 2 are myokines linking muscle to bone in response to gravity changes and mechanical unloading in mice [14–16]. We also demonstrated that irisin, a proteolytic product of fibronectin type III domain-containing 5 (Fndc5), in skeletal muscle is related to bone loss during unloading and exerts protective effects of chronic exercise on estrogen deficiency-induced bone loss in mice [17, 18]. Recently, we showed that renal failure decreases the expression of irisin in the gastrocnemius muscles of mice and that irisin contributes to cortical bone loss induced by renal failure in mice as a myokine linking muscle to bone [19]. However, the effects of electrolyte abnormalities, such as hyponatremia, on myokines linking muscle to bone have not been reported, although hyponatremia induces both sarcopenia and osteoporosis.

In the present study, we aimed to examine the effects of hyponatremia on muscle and bone as well as myokines linking muscle to bone using two hyponatremia mouse models, such as 1-desamino-8-D-arginine vasopressin (dDAVP)- and furosemide-treated mice with hyponatremia complicated by increased and decreased extracellular fluid in the body, respectively.

## Materials and Methods

### Animals and Hyponatremia Models

Male C57BL/6J mice were obtained from CLEA Japan (Tokyo, Japan). For the experiments involving the administration of dDAVP, mice were divided into 2 groups: control group (n = 8) and dDAVP group (n = 8). dDAVP (Selleck Biotech, Tokyo, Japan) was administered to 9-week-old mice for 8 weeks as previously described [20]. Briefly, osmotic minipumps (Alzet model 1004; Durect Corporation, Cupertino, CA) containing dDAVP solution were implanted subcutaneously into mice under 2% isoflurane anesthesia. The injection rate of dDAVP was 0.5 ng/h. One day after dDAVP administration, mice were loaded water by substituting their daily feed with a gelled food containing 72% water, 27% finely ground mouse chow, and 1% agar [21]. In experiments involving the administration of furosemide, male mice were divided into 2 groups: control (n = 6) and furosemide (n = 5). Mice were injected subcutaneously with 250 mg/kg furosemide (Tokyo Kasei Kogyo, Tokyo, Japan) four times per week from 9 weeks of age for 8 weeks. In experiments involving the administration of furosemide and sodium chloride (NaCl), male mice were divided into 4 groups: water/control (n = 8), NaCl/control (n = 8), water/furosemide (n = 8), and NaCl/furosemide (n = 8). Mice were injected subcutaneously with 250 mg/kg furosemide as described above. NaCl was administered via drinking water at a concentration of 10 g/L for 8 weeks after the first injection of furosemide. Mice accessed food and water ad libitum. Eight weeks after the first injection of dDAVP or furosemide, mice were euthanized with excess isoflurane and tissue samples were collected and weighed.

Animal experiments were performed in accordance with the guidelines of ARRIVE and the institutional rules for the use and care of laboratory animals at Kindai University. All experimental procedures on animals were approved by the Experimental Animal Welfare Committee of Kindai University (Permit Number: KAME-2022-079).

### Blood Chemistry

Blood samples were obtained from mice via cardiac puncture and centrifuged at 1000×*g* at 4 °C for 15 min after clotting. Serum levels of sodium were analyzed by Oriental Kobo Co. Ltd. (Tokyo, Japan). Serum levels of IGF-1 were measured using Mouse IGF-1 ELISA Kit (Cat. No. EMI1001-1, Assaypro LLC, St. Charles, MO) in accordance with the manufacturers’ instructions.

### Measurement of Grip Strength

Grip strength of four limbs was measured using a grip strength meter (1027SM, Columbus Instruments, Columbus, OH, USA) 8 weeks after the first injection of dDAVP or furosemide with or without NaCl administration, as previously described [19].

### Quantitative Computed Tomography (QCT)

Mice were scanned 8 weeks after the first injection of dDAVP or furosemide using an X-ray CT system in vivo (Latheta LCT-200; Hitachi Aloka Medical, Tokyo, Japan) as previously described [19]. After mice were anesthetized with 2% isoflurane, CT images were acquired using the following parameters: tube voltage, 50 kVp; tube current, 500 µA; axial field of view, 48 mm; voxel size, 96 × 192 × 1008 µm for analyses of muscle mass in the whole body; and 48 × 48 × 192 µm voxel size to analyze muscle mass in the lower legs. Regions of interest were defined as the whole body for the assessment of muscle mass in the whole body. Regions of interest for the assessment of muscle mass in the lower legs were defined as the segment from the proximal to distal end of the tibia. QCT data were analyzed using LaTheta software (version 3.41).

### Micro-computed Tomography (μCT)

A μCT analysis was performed according to our previous study [22] and the guidelines of the American Society for Bone and Mineral Research [23]. Mice were scanned 8 weeks after the first administration of dDAVP or furosemide. The distal metaphyseal regions of the femurs were scanned using CosmoScan GXII (Rigaku Corporation, Yamanashi, Japan) with the following parameters: voxel size = 10 × 10 × 10 μm; X-ray voltage = 90 kV; X-ray tube current = 88 µA; and exposure time = 4 min. Beam-hardening artifacts were reduced using copper (0.06 mm) and aluminum (0.5 mm) filters. Prior to the analysis of the bone microstructure, raw images were reconstructed with CosmoScan GX Image Analysis Software (Rigaku Corporation) with an isotropic voxel size of 5.5 µm. The microstructural parameters of the femurs were assessed using Analyze 14.0 software (AnalyzeDirect, Inc., KS, USA). A 1-mm-thick region from the end of the growth plate was used in the trabecular bone analysis, and the following parameters were assessed: trabecular BMD, bone volume fraction (BV/TV), trabecular number (Tb.N), trabecular thickness (Tb.Th), and trabecular separation (Tb.Sp). A 1-mm-thick region of the mid-diaphysis of the femur was used for cortical bone analysis, and the following parameters were assessed: cortical BMD, cortical area (Ct.Ar), and cortical thickness (Ct.Th).

### Western Blot

A Western blot analysis was performed with anti-phosphorylated Akt at Ser473 (Cat. No. 4060, Cell Signaling Technology, Danvers, MA, USA), anti-Akt (Cat. No. 4691, Cell Signaling Technology), anti-phosphorylated p70 S6 kinase at Thr389 (Cat. No. 9234, Cell Signaling Technology), anti-p70 S6 kinase (Cat. No. 2708, Cell Signaling Technology), anti-IGF-1 (Cat. No. bs-0014R, Bioss Antibodies, Beijing, China) and anti-glyceraldehyde-3-phosphate dehydrogenase (GAPDH) antibodies (Cat. No. 5174, Cell Signaling Technology) as described previously [19].

### Real-time Polymerase Chain Reaction (PCR)

A real-time PCR analysis was performed using QuantStuio 7 Flex Real-Time PCR System (Applied Biosystems, Foster, CA, USA) and Fast SYBR Green Master Mix (Applied Biosystems) as previously described [19]. PCR primer sets are shown in Table S1. The specific mRNA amplification of the target gene was normalized to 18S ribosomal RNA levels and analyzed using the ΔΔCt method.

### Cell Culture

Mouse myoblastic C2C12 cells (ATCC, Manassas, VA, USA) were cultured in Dulbecco’s Modified Eagle’s Medium (DMEM; Wako Pure Chemicals) supplemented with 10% fetal bovine serum (FBS), 100 units/ml penicillin, and 100 μg/ml streptomycin. Subconfluent C2C12 cells were cultured in DMEM supplemented with 2% horse serum for 5 days to facilitate myogenic differentiation. To generate low-sodium culture medium, DMEM was diluted with sterile Milli-Q water containing normal concentrations of minerals except sodium, amino acids, and vitamins (Wako Pure Chemicals) with 10% FBS, 100 units/ml penicillin, and 100 μg/ml streptomycin. The osmolality of the low-sodium culture medium was adjusted to 300 mOsm/L with mannitol. The sodium levels and osmolality of the culture medium were analyzed by Oriental Kobo Co. Ltd.

### Statistical Analysis

Each experiment was performed at least three times. The data are expressed as the mean ± the standard error of the mean (SEM). The significance of differences was evaluated using the Mann‒Whitney U test for two group comparisons and a two-way analysis of variance followed by the Tukey‒Kramer test for multiple comparisons. A simple regression analysis was performed using Pearson’s test. The significance of differences was defined as *P* < 0.05. Statistical analyses were performed using GraphPad PRISM 7.00 software (GraphPad Software, La Jolla, CA, USA).

## Results

### Effects of Hyponatremia on Muscle and Bone in Mice

**Fig. 1:**
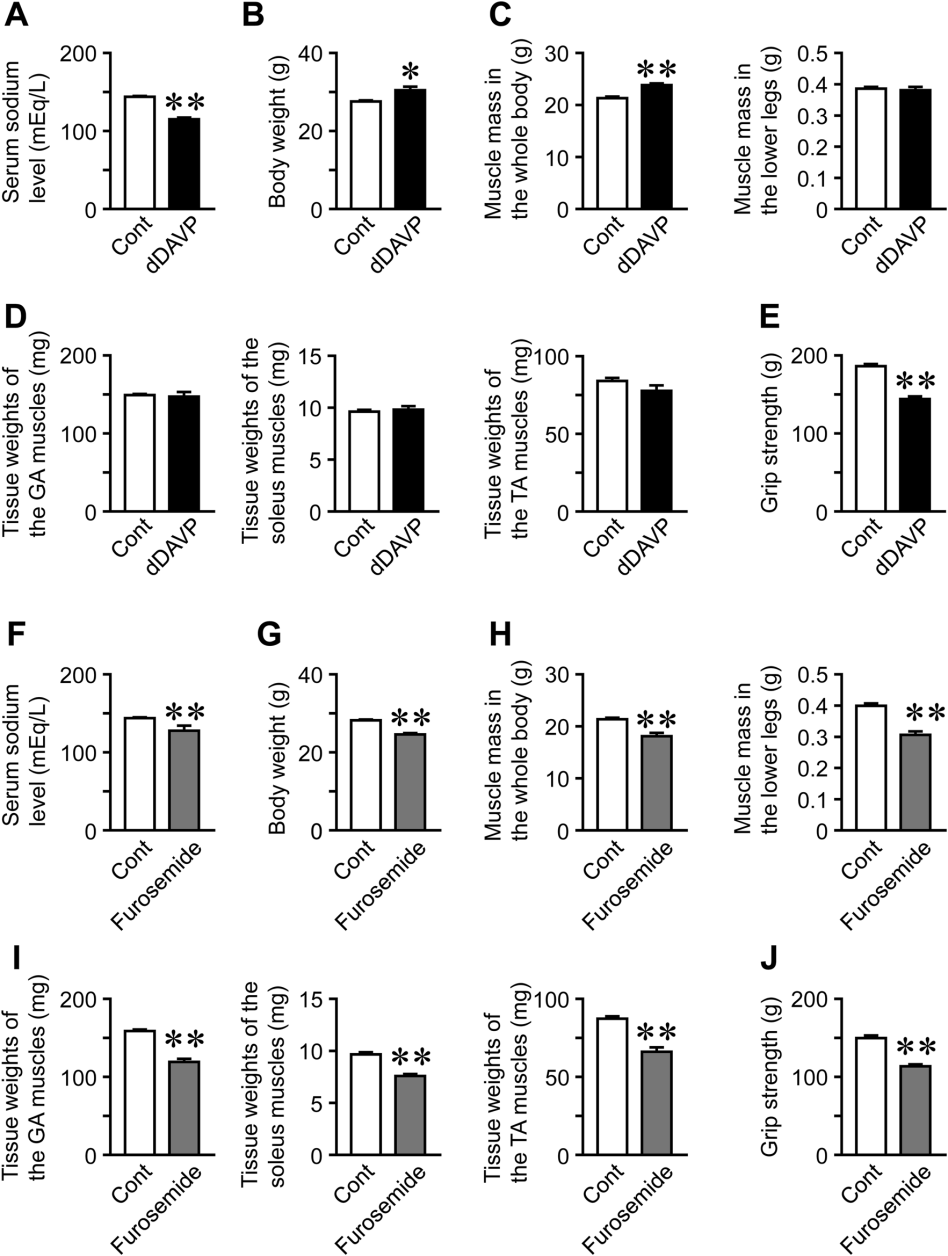
Effects of hyponatremia on muscle. **A**, **F** Serum samples were collected from mice 8 weeks after the first injection of dDAVP (**A**) or furosemide (**F**). Serum sodium levels were analyzed. **B**, **G** Data on body weight in control and hyponatremic mice. Body weight was measured 8 weeks after the first injection of dDAVP (**B**) or furosemide (**G**). **C**, **H** Muscle mass in the whole body and lower limbs were assessed by QCT 8 weeks after the first injection of dDAVP (**C**) or furosemide (**H**). **D**, **I** The gastrocnemius (GA), soleus, and tibial anterior (TA) muscles of mice were weighed 8 weeks after the first injection of dDAVP (**D**) or furosemide (**I**). **E**, **J** The grip strength of the four limbs was measured using a grip strength meter 8 weeks after the first injection of dDAVP (**E**) or furosemide (**J**). Data represent the mean ± SEM (n = 8 mice in dDAVP and its control groups, n = 5 mice in furosemide and 6 mice in its control groups). **P < 0.01 and *P < 0.05

**Fig. 2:**
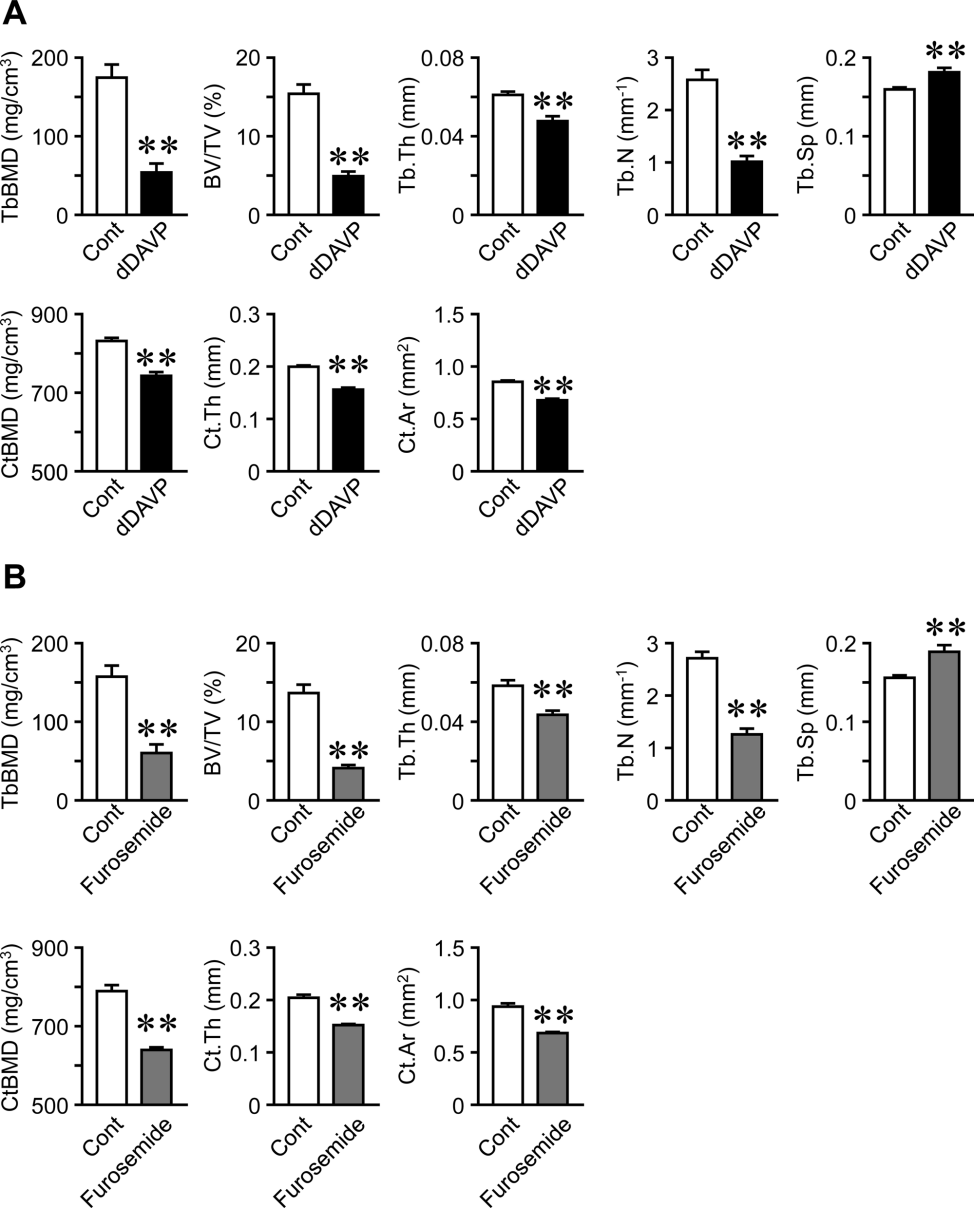
Effects of hyponatremia on bone. **A**, **B** Trabecular BMD (TbBMD), trabecular bone volume fraction (BV/TV), trabecular thickness (Tb.Th), trabecular number (Tb.N), trabecular separation (Tb.Sp), cortical BMD (CtBMD), cortical thickness (Ct.Th), and cortical area (Ct.Ar) at the femurs of mice were assessed by µCT 8 weeks after the first injection of dDAVP (**A**) or furosemide (**B**). Data represent the mean ± SEM (n = 8 mice in dDAVP and its control groups, n = 5 mice in furosemide and 6 mice in its control groups). **P < 0.01 and *P < 0.05

dDAVP and furosemide significantly reduced serum sodium levels in mice (Fig. 1A, F), which indicated the successful induction of hyponatremia in these mouse models. Body weight and muscle mass in the whole body were significantly higher in dDAVP mice than in control mice; however, dDAVP did not affect muscle mass in the lower legs, or tissue weights of the gastrocnemius, soleus, or tibial anterior muscles (Fig. 1B–D). dDAVP significantly decreased grip strength in mice (Fig. 1E). Furosemide significantly reduced body weight, muscle mass in the whole body and lower legs, tissue weights of the gastrocnemius, soleus, and tibial anterior muscles, and grip strength in mice (Fig. 1F–J). As for bone, trabecular BMD, trabecular bone volume (BV/TV), trabecular thickness, trabecular number, cortical BMD, cortical thickness, and cortical area at the femur were significantly lower in dDAVP- or furosemide-treated mice than in each control mice, although dDAVP and furosemide significantly increased trabecular separation (Fig. 2A, B).

### Effects of Hyponatremia on Muscle Protein Synthesis Signaling and Degradation-Related Gene Expression

**Fig. 3:**
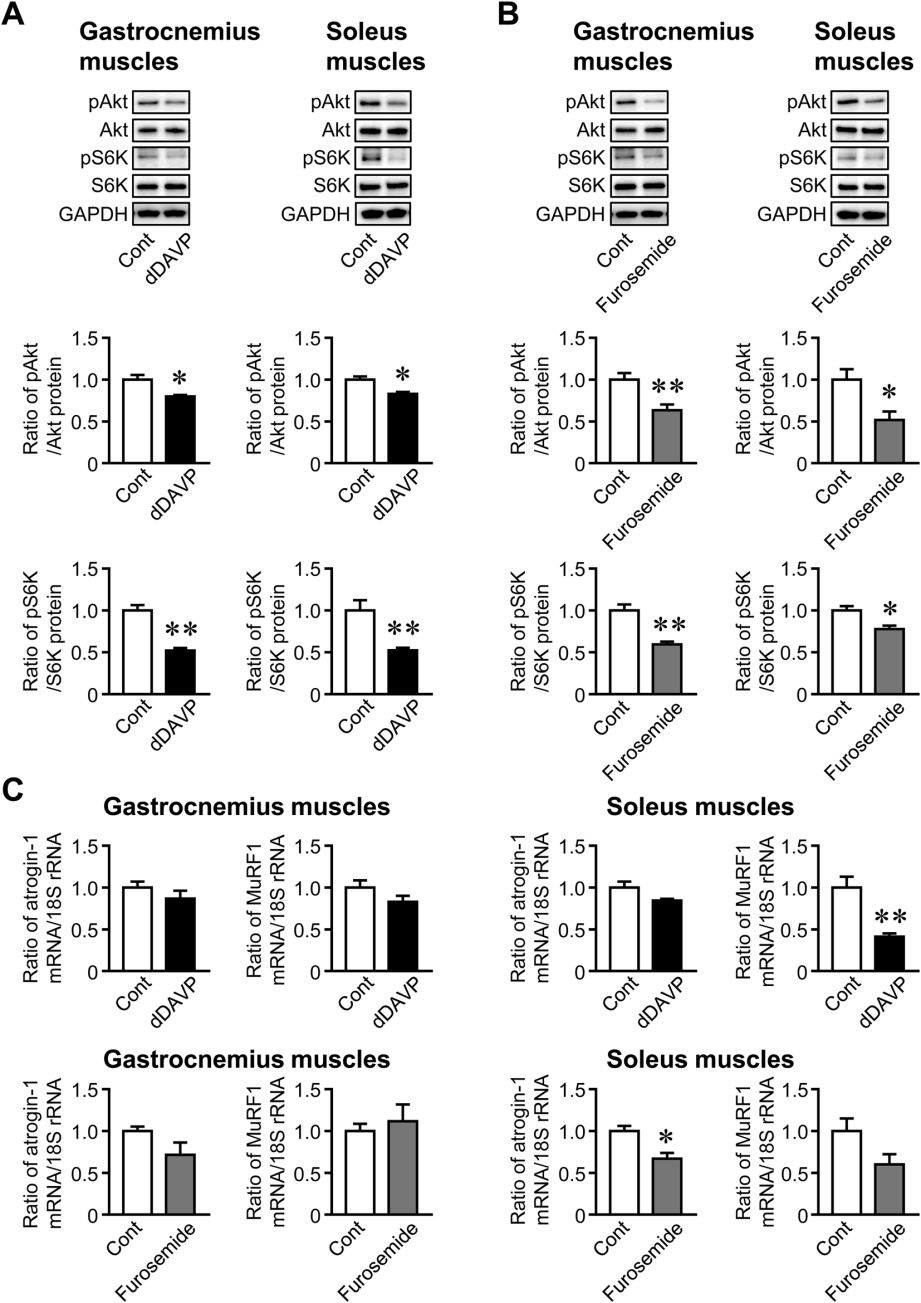
Effects of hyponatremia on protein synthesis signaling and degradation-related gene expression. **A**, **B** Total proteins were extracted from the gastrocnemius and soleus muscles of mice 8 weeks after the first injection of dDAVP (**A**) or furosemide (**B**). Western blot analyses of phosphorylated Akt (pAkt), Akt, phosphorylated p70 S6 kinase (pS6K), S6K, and GAPDH were performed. Images represent experiments performed independently at least three times. Protein levels of IGF-1 were quantified by densitometry and adjusted by GAPDH levels. Data represent the mean ± SEM (n = 5 in each group). **C** Total RNA was extracted from the gastrocnemius and soleus muscles of mice 8 weeks after the first injection of dDAVP or furosemide. A real-time PCR analysis of atrogin-1, MuRF1, and 18S rRNA was performed. Data represent the mean ± SEM (n = 8 mice in dDAVP and its control groups, n = 5 mice in furosemide and 6 mice in its control groups). **P < 0.01 and *P < 0.05

dDAVP and furosemide significantly decreased phosphorylation of Akt and p70 S6 kinase, a crucial protein synthesis pathway, in the gastrocnemius and soleus muscles of mice (Fig. 3A, B). dDAVP and furosemide significantly reduced the mRNA levels of MuRF1 and atrogin-1, E3 ubiquitin ligases, respectively, in the soleus muscles of mice, but did not affect those mRNA levels in the gastrocnemius muscles (Fig. 3C).

### Effects of Hyponatremia on Myokine Expression in the Gastrocnemius and Soleus Muscles

**Fig. 4:**
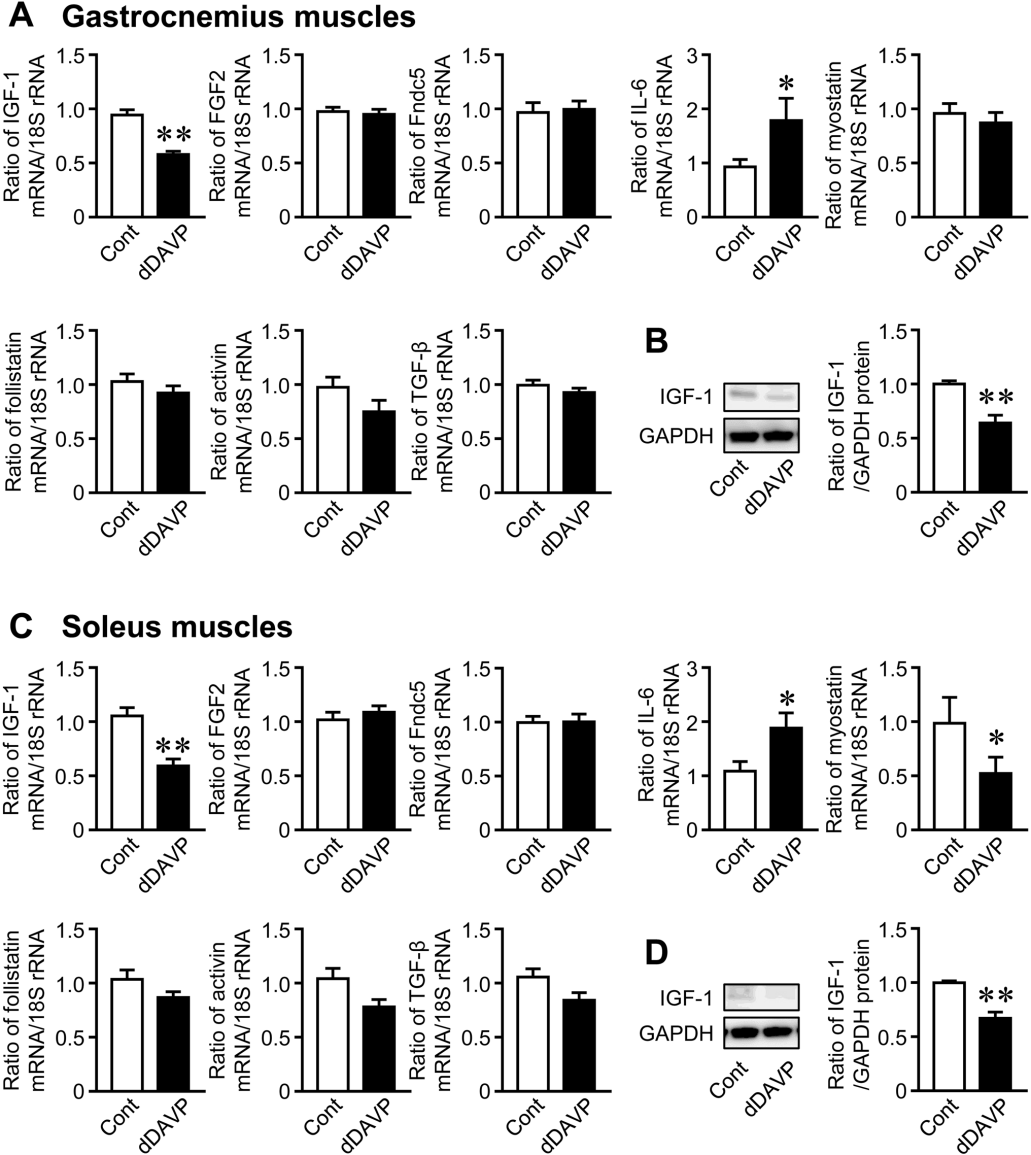
Effects of dDAVP-induced hyponatremia on the expression of myokines. **A**, **C** Total RNA was extracted from the gastrocnemius (**A**) and soleus (**C**) muscles of mice 8 weeks after the first injection of dDAVP. A real-time PCR analysis of IGF-1, FGF2, Fndc5, IL-6, myostatin, follistatin, activin, TGF-β, and 18S rRNA was performed. Data represent the mean ± SEM (n = 8 mice in each group). **P < 0.01 and *P < 0.05. **B**, **D** Total proteins were extracted from the gastrocnemius (**B**) and soleus (**D**) muscles of mice 8 weeks after the first injection of dDAVP. Western blot analyses of IGF-1 and GAPDH were performed. Images represent experiments performed independently at least three times. Protein levels of IGF-1 were quantified by densitometry and adjusted by GAPDH levels. Data represent the mean ± SEM (n = 5 in each group). **P < 0.01 and *P < 0.05

**Fig. 5:**
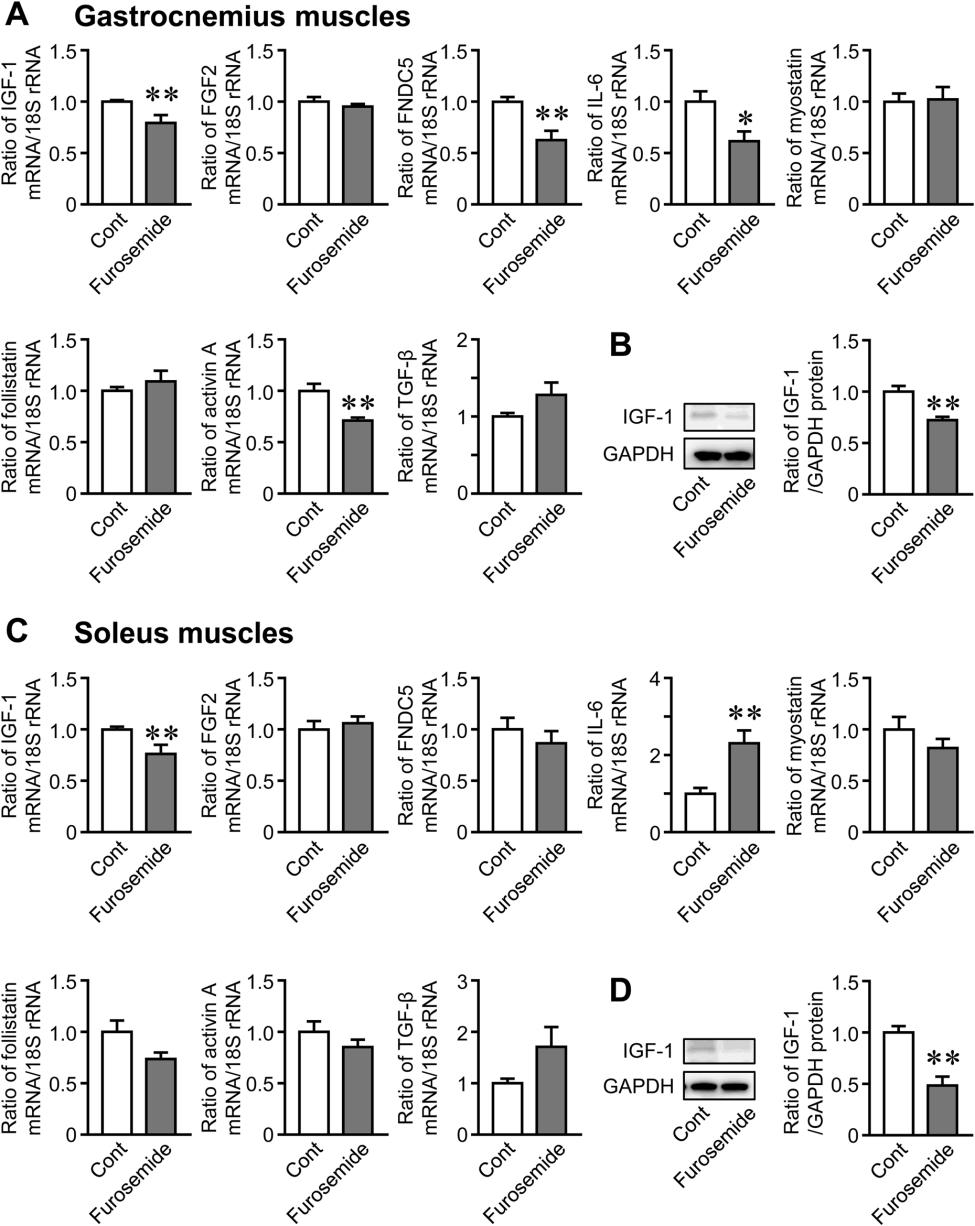
Effects of furosemide-induced hyponatremia on the expression of myokines. **A**, **C** Total RNA was extracted from the gastrocnemius (**A**) and soleus (**C**) muscles of mice 8 weeks after the first injection of furosemide. A real-time PCR analysis of IGF-1, FGF2, Fndc5, IL-6, myostatin, follistatin, activin, TGF-β, and 18S rRNA was performed. Data represent the mean ± SEM (n = 6 mice in control and 5 mice in furosemide groups). **P < 0.01 and *P < 0.05. **B**, **D** Total proteins were extracted from the gastrocnemius (**B**) and soleus (**D**) muscles of mice 8 weeks after the first injection of furosemide. Western blot analyses of IGF-1 and GAPDH were performed. Images represent experiments performed independently at least four times. Protein levels of IGF-1 were quantified by densitometry and adjusted by GAPDH levels. Data represent the mean ± SEM (n = 4 in each group). **P < 0.01 and *P < 0.05

**Fig. 6:**
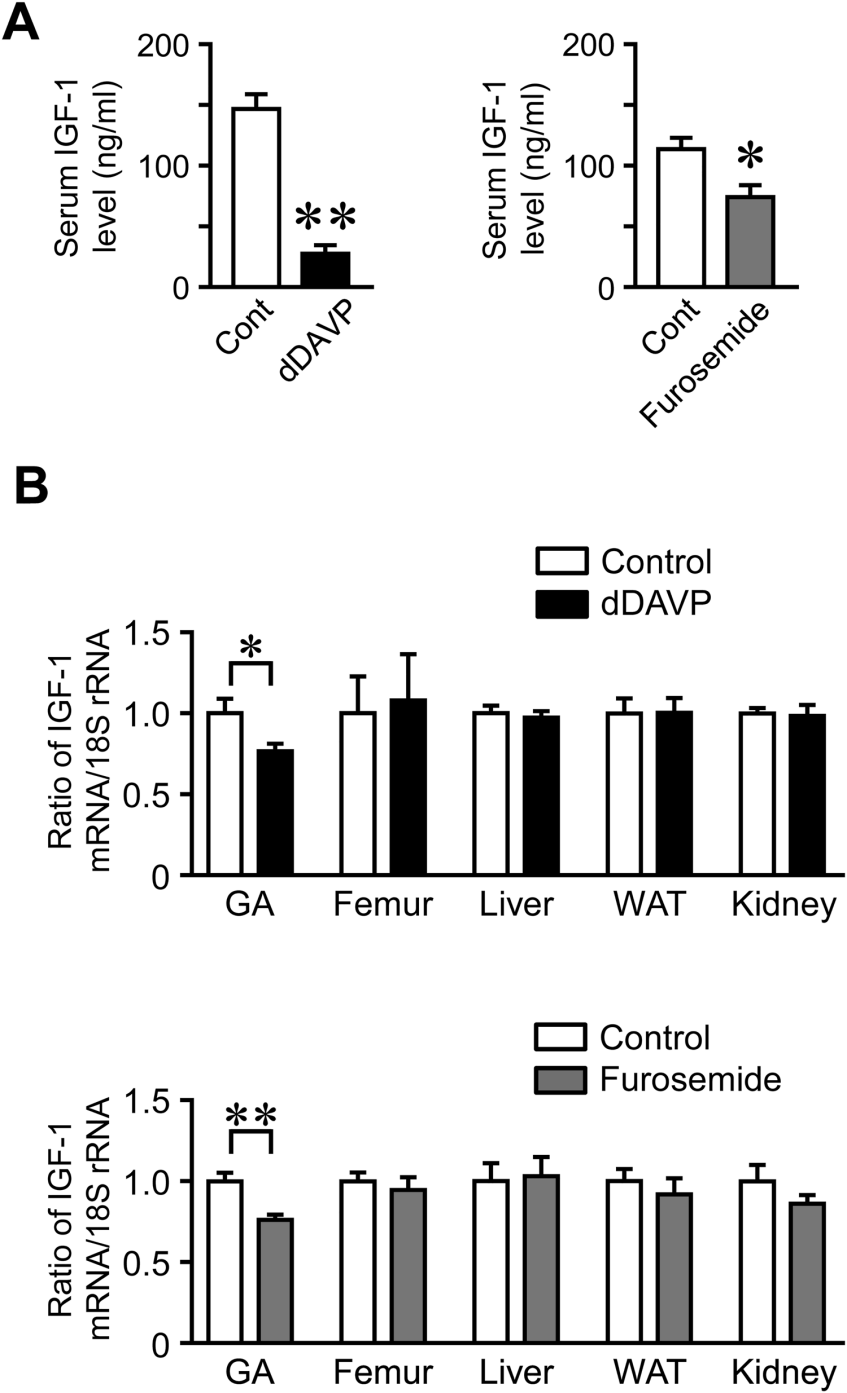
Effects of hyponatremia on the circulating IGF-1 levels and expression of IGF-1 in mouse tissues. **A** Serum samples were collected from mice 8 weeks after the first injection of dDAVP or furosemide. Serum IGF-1 levels were analyzed. **P < 0.01 and *P < 0.05. **B** Total RNA was extracted from the gastrocnemius muscles (GA), femurs, liver, visceral white adipose tissue (WAT), and kidneys of mice 8 weeks after the first injection of dDAVP or furosemide. A real-time PCR analysis of IGF-1 and 18S rRNA was performed. Data represent the mean ± SEM (n = 8 mice in dDAVP and its control groups, n = 5 mice in furosemide and 6 mice in its control groups)

dDAVP significantly decreased the mRNA and protein levels of IGF-1 in the gastrocnemius and soleus muscles of mice (Fig. 4A–D). It also significantly increased IL-6 mRNA levels in the gastrocnemius and soleus muscles (Fig. 4A, C). dDAVP significantly reduced myostatin mRNA levels in the soleus muscles, but did not affect the mRNA levels of FGF2, Fndc5, follistatin, activin, or TGF-β (Fig. 4C). Furosemide significantly reduced the mRNA and protein levels of IGF-1 in the gastrocnemius and soleus muscles of mice (Fig. 5A–D). Furosemide significantly decreased and increased IL-6 mRNA levels in the gastrocnemius and soleus muscles, respectively (Fig. 5A, C). It also reduced the mRNA levels of Fndc5 and activin in the gastrocnemius muscles, but did not affect the mRNA levels of FGF2, myostatin, follistatin, or TGF-β in the gastrocnemius or soleus muscles (Fig. 5A, C). dDAVP and furosemide significantly reduced serum IGF-1 levels in mice (Fig. 6A). We then investigated the expression of IGF-1 in various tissues. dDAVP and furosemide reduced IGF-1 mRNA levels in the gastrocnemius muscle but not in the femurs, liver, white adipose tissues, or kidneys of mice (Fig. 6B).

### Relationships Between IGF-1 Levels and Muscle or Bone Parameters in Mice

We investigated the relationships between IGF-1 levels and muscle or bone parameters in mice using simple regression analyses. The IGF-1 mRNA levels in the gastrocnemius and soleus muscles were significantly and positively related to trabecular bone volume, cortical BMD, and grip strength in mice (Table S2). Serum IGF-1 levels were significantly and positively related to trabecular bone volume, cortical BMD, and grip strength in mice (Table S3). Serum IGF-1 levels were significantly and positively related to the IGF-1 mRNA levels in the gastrocnemius and soleus muscles of mice (Table S3).

### Effects of Low Sodium Concentrations on IGF-1 Expression in Mouse C2C12 Cells

**Fig. 7:**
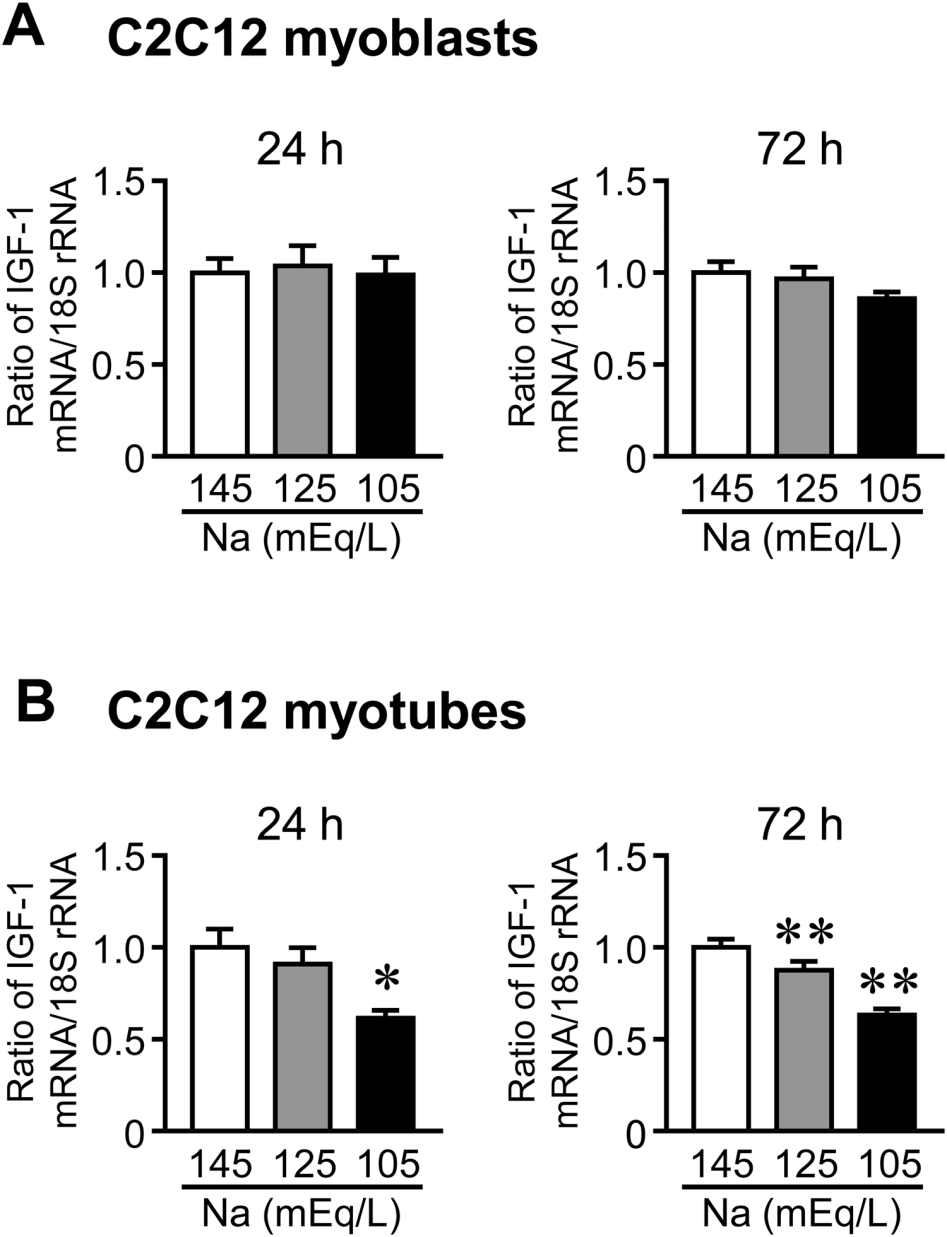
Effects of low sodium concentrations on IGF-1 expression in C2C12 cells. **A**, **B** Total RNA was extracted from C2C12 myoblasts (**A**) and myotubes (**B**) 24 or 72 h after culture in medium containing indicated concentrations of sodium, and real-time PCR analysis of IGF-1 and 18S rRNA was performed. Data represent the mean ± SEM (n = 5 sample in each group). Cont; Control. **P < 0.01 and *P < 0.05

Next, we examined the effects of hyponatremia on IGF-1 expression in mouse C2C12 cells. Low sodium concentrations significantly reduced IGF-1 mRNA levels in C2C12 myotubes but not in myoblasts (Fig. 7A, B).

### Effects of NaCl Administration on Muscle and Bone in Mice with Hyponatremia

**Fig. 8:**
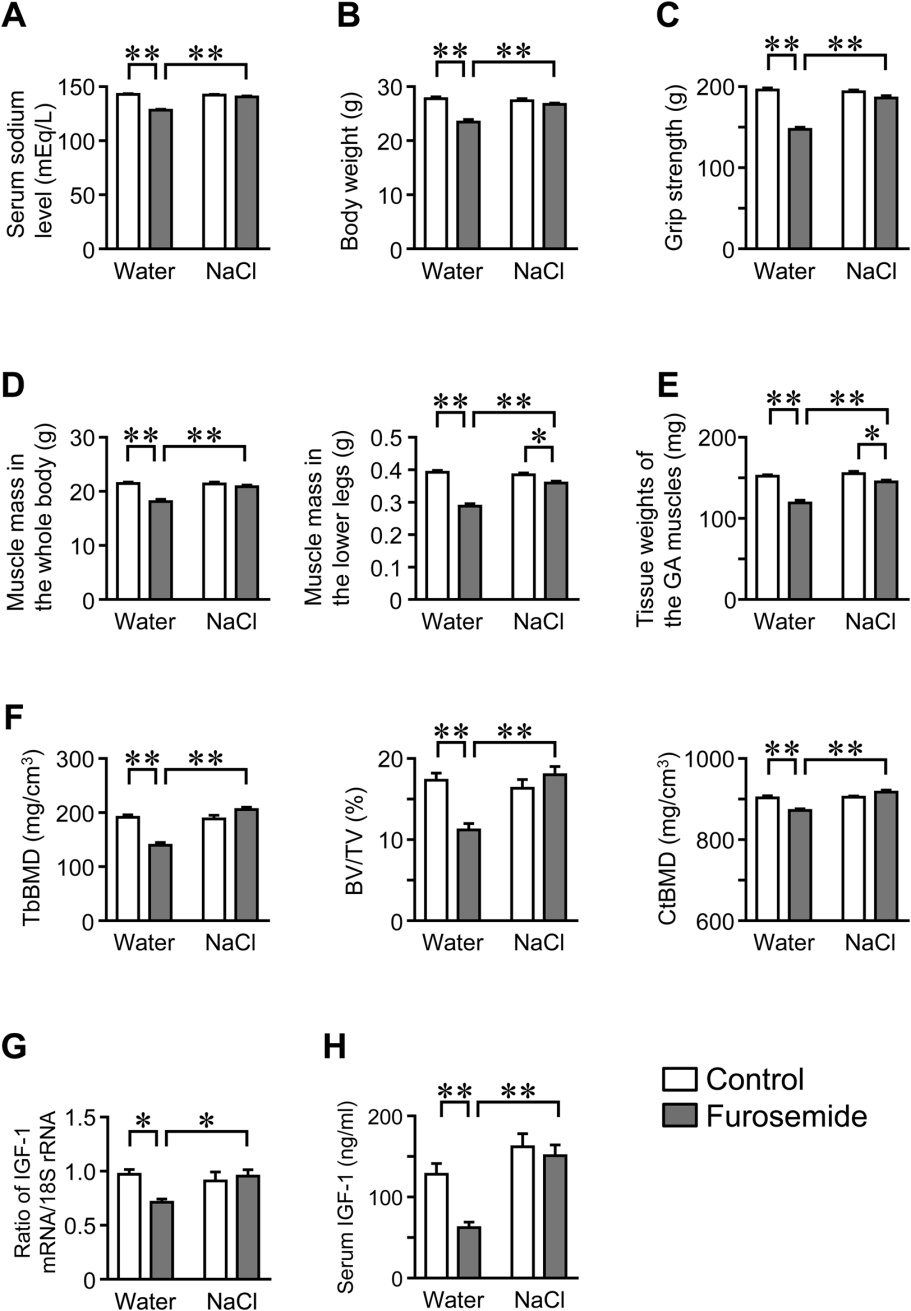
Effects of NaCl administration on muscle and bone in furosemide-induced hyponatremic mice. **A** Serum samples were collected from mice 8 weeks after the first injection of furosemide with or without NaCl administration. Serum sodium levels were analyzed. **B** Body weight was measured 8 weeks after the first injection of furosemide with or without NaCl administration. **C** The grip strength of the four limbs was measured using a grip strength meter in mice 8 weeks after the first injection of furosemide with or without NaCl administration. **D** Muscle mass in the whole body and lower limbs were assessed by QCT 8 weeks after the first injection of furosemide with or without NaCl administration. **E** The gastrocnemius (GA) muscles of mice were weighed 8 weeks after the first injection of furosemide with or without NaCl administration. **F** Trabecular BMD (TbBMD), trabecular bone volume fraction (BV/TV), and cortical BMD (CtBMD) at the femurs of mice were assessed by µCT 8 weeks after the first injection of furosemide. **G** Total RNA was extracted from the gastrocnemius muscles of mice 8 weeks after the first injection of furosemide with or without NaCl administration. A real-time PCR analysis of IGF-1 and 18S rRNA was performed. Data are expressed relative to the levels of 18S rRNA. **H** Serum IGF-1 levels were analyzed 8 weeks after the first injection of furosemide with or without NaCl administration. Data represent the mean ± SEM. n = 8 mice in each group. Cont; Control. *P < 0.05 and **P < 0.01

The administration of NaCl did not affect serum sodium levels, body weight, grip strength, muscle mass in the whole body or lower legs, tissue weights of the gastrocnemius muscles, trabecular BMD, trabecular bone volume, cortical BMD, or IGF-1 mRNA levels in the gastrocnemius muscles, or serum IGF-1 levels in control mice (Fig. 8A–H). On the other hand, the administration of NaCl significantly improved furosemide-induced reductions in serum sodium levels, body weight, grip strength, muscle mass in the whole body and lower legs, tissue weights of the gastrocnemius muscles, trabecular BMD, trabecular bone volume, cortical BMD, IGF-1 mRNA levels in the gastrocnemius muscles, and serum IGF-1 levels (Fig. 8A–H).

## Discussion

In the present study, we showed that the administration of dDAVP and furosemide caused hyponatremia followed by decreases in bone mass and grip strength in mice. Furosemide significantly decreased muscle mass and tissue weights of the gastrocnemius, soleus, and tibial anterior muscles of mice. The administration of dDAVP and furosemide significantly reduced serum levels of IGF-1 and muscle IGF-1 expression, which were positively related to trabecular bone volume and cortical BMD at the femurs in simple regression analyses of mouse samples employed in our study. Moreover, we revealed that NaCl treatment ameliorated furosemide-induced reductions in muscle and bone parameters as well as serum IGF-1 levels and muscle IGF-1 expression in mice.

We showed that the administration of dDAVP significantly decreased grip strength in mice. Moreover, furosemide significantly reduced grip strength, muscle mass, and tissue weights of the gastrocnemius, soleus, and tibial anterior muscles in mice, which was consistent with the findings of a previous study [10]. In addition, NaCl treatment blunted these effects of furosemide on skeletal muscles of mice. Although serum sodium levels were decreased at concentrations of 120–130 mEq/L by the administration of dDAVP or furosemide in mice, a clinical study revealed that mild hyponatremia at 130–135 mEq/L serum sodium is related to decreased skeletal muscle mass and impaired physical function in elderly individuals [11]. Taken together, our data suggest that hyponatremia reduces muscle mass and strength in mice. However, dDAVP significantly increased body weight and whole-body muscle mass, as assessed by qCT, and did not affect the tissue weights of the gastrocnemius, soleus, or tibial anterior muscles of mice. Since the volume of extracellular body fluid is increased by the administration of dDAVP, we speculated that the changes in muscle mass and tissue weights of skeletal muscles induced by dDAVP might be masked by the increase in the amount of extracellular body fluid in dDAVP-treated mice.

Numerous studies have revealed that skeletal muscles release a variety of humoral factors that affect local and distant organs [13, 24]. We showed that the administration of dDAVP or furosemide changes the expression of myokines, including IGF-1, IL-6, myostatin, irisin, and activin in the gastrocnemius or soleus muscles of mice, suggesting that hyponatremia affects muscle secretome in mice. In the present study, both dDAVP and furosemide significantly suppressed serum IGF-1 levels and IGF-1 expression in the gastrocnemius and soleus muscles of mice compared with those in the control mice. Moreover, NaCl treatment attenuated the decrease in serum IGF-1 levels and IGF-1 expression in the gastrocnemius muscles caused by furosemide in mice. These data indicate that hyponatremia suppresses IGF-1 expression and secretion in the skeletal muscles of mice.

Circulating IGF-1 is released mainly from the liver and exerts growth effects in various tissues. IGF-1 is also expressed in almost all tissues and is locally affected in a paracrine and autocrine manner [25]. Previous studies revealed that IGF-1 contributes to muscle growth by promoting myoblast differentiation and proliferation [25, 26]. IGF-1 increases muscle mass by enhancing and suppressing protein synthesis and degradation, respectively, in skeletal muscles through activation of the phosphatidylinositol 3-kinase (PI3K)/Akt pathway [25]. Although IGF-1 is involved in the effects of aging, nutritional abnormalities, and exercise on muscle and bone [25–30], the present findings suggest that decreased IGF-1 expression in skeletal muscles might contribute to the hyponatremia-induced decrease in muscle mass in mice. Analysis of cell‒cell interactions in mononuclear cells from porcine skeletal muscles using single-cell RNA sequencing revealed that satellite cells communicate with fibro-adipogenic progenitors (FAPs) via the release of IGF-1 in fast-twitched myofiber-dominant porcine skeletal muscles [31]. Wosczyna et al. revealed that conditional deletion of FAPs in adult mice induces muscle fiber atrophy, suggesting that FAPs are involved in the maintenance of muscle mass under physiological conditions [32]. We therefore speculated that hyponatremia might impair the interactions among cells composed of skeletal muscle tissues via IGF-1, leading to muscle atrophy in mice.

In the present study, the administration of dDAVP or furosemide decreased grip strength in mice, which was consistent with previous clinical reports [11, 12]. Moreover, NaCl administration attenuated the decrease in grip strength caused by furosemide in mice. These findings indicate that a hyponatremic state reduces muscle strength in mice. IGF-1 mRNA levels in the gastrocnemius and soleus muscles were positively correlated with grip strength in simple regression analyses of the mice used in the experiments. Van Nieuwpoort et al. reported that lower serum IGF-1 levels are associated with lower handgrip strength and worse physical performance [33]. Taken together, decreased IGF-1 levels in skeletal muscles might be partly associated with diminished muscle strength induced by hyponatremia.

In the present study, the administration of dDAVP and furosemide significantly reduced trabecular bone volume as well as trabecular and cortical BMD of the femurs of mice. Moreover, NaCl treatment attenuated the furosemide-induced decreases in these bone parameters in mice. These data are consistent with previous reports [2, 10]. Taken together, our findings suggest that hyponatremia induces bone loss in mice. Skeletal muscle interacts with bone through humoral factors, such as myokines. We showed that serum IGF-1 levels and IGF-1 expression in skeletal muscles were positively related to bone parameters in mice. As for bone, a previous report showed that IGF-1 increased bone mass and enhanced osteoblastic bone formation in mice [25]. Moreover, IGF-1 facilitates osteogenic differentiation and bone morphogenetic protein-2-induced matrix mineralization in mesenchymal stem cells [34]. Numerous studies have indicated that IGF-1 stimulates survival, proliferation, differentiation, and production of bone matrix proteins in osteoblasts [25]. Yakar et al. reported that a threshold concentration of circulating IGF-1 is necessary for normal bone growth in mice [35]. Taken together, these findings suggest that the decreases in circulating and muscle-derived IGF-1 contribute to the hyponatremia-induced bone loss in mice. IGF-1 might be involved in as a myokine linking muscle to bone in hyponatremia-induced osteoporosis in mice.

In the present study, IGF-1 expression in muscle was decreased in mice with hyponatremia. Although approximately 75% of circulating IGF-1 is produced by the liver, liver-derived IGF-1 exerts a small effect on cortical bone but does not affect trabecular bone, as assessed in liver-specific IGF-1-deficient mice [36]. On the other hand, muscle-specific depletion of GRP94, which is a crucial factor for IGF-1 production, resulted in a shorter femur bone length than that in wild-type mice [37]. Moreover, ectopic expression and overexpression of IGF-1 in skeletal muscle inhibited unloading-induced bone loss and increased bone mass, respectively, in mice [38]. Collectively, these findings suggest a possibility that decreased IGF-1 levels in skeletal muscle might be a responsible for the hyponatremia-induced bone loss in mice. However, we cannot exclude that decreased muscle mass and strength caused by decreased IGF-1 expression in skeletal muscle might be related to bone loss due to a decrease in mechanical stress in hyponatremic mice.

Increasing clinical evidence suggests that sarcopenia and osteoporosis are related to each other. However, there are currently no common biomarkers for sarcopenia or osteoporosis. The present results raised a possibility that muscle-derived IGF-1 might contribute to hyponatremia-induced muscle and bone loss in mice. Therefore, IGF-1 may be expected to be a biomarker for hyponatremia-induced sarcopenia and bone loss, although there is limitation that serum IGF-1 level is influenced by IGF-1 derived from organs other than skeletal muscles.

There are several limitations in this study. First, we could not rule out the possibility that the data of the in vivo experiments might be partly due to the pharmacological effects of dDAVP or furosemide other than the effects of hyponatremia. Second, further studies using skeletal muscle-specific IGF-1-deficient mice are necessary to precisely clarify the role of muscle-derived IGF-1 in hyponatremia-induced osteopenia in mice. Third, the mechanisms by which hyponatremia reduces IGF-1 expression in skeletal muscles have still remained unknown in the present study.

In conclusion, we herein demonstrated that hyponatremia reduces muscle mass, muscle strength, and bone mass accompanied with decreases in muscle and circulating IGF-1 levels in mice. The present findings suggest that a decrease in IGF-1 expression in skeletal muscle is related to bone loss induced by hyponatremia in mice.

## Supplementary Information

Below is the link to the electronic supplementary material.

Supplementary file1 (DOCX 16 KB)

Supplementary file2 (DOCX 18 KB)

Supplementary file3 (DOCX 17 KB)

## Data Availability

The datasets generated during and/or analyzed during the current study are not publicly available but are available from the corresponding author on reasonable request.
