# Supplementary material for: Roles of Insulin-Like Growth Factor-1 in Muscle Wasting and Osteopenia in Mice with Hyponatremia
Source: Calcif Tissue Int. 2025 Apr 14;116(1):61. doi: 10.1007/s00223-025-01369-7 (PMC11996959; doi:10.1007/s00223-025-01369-7)
Supplement: Supplementary file 1 — Supplementary file1 (DOCX 16 KB) [file 223_2025_1369_MOESM1_ESM.docx]

**Table S1** Primers used for real-time PCR experiments.

| Gene |  | Primer sequence |
| --- | --- | --- |
| Atrogin-1  MuRF1  IGF-1 | Forward  Reverse  Forward  Reverse  Forward  Reverse | 5’-GTCGCAGCCAAGAAGAGAAAGA-3’  5’-TGCTATCAGCTCCAACAGCCTT-3’  5’-TAACTGCATCTCCATGCTGGTG-3’  5’-TGGCGTAGAGGGTGTCAAACTT-3’  5’-CAAGCCCACAGGCTATGGC-3’  5’-TCTGAGTCTTGGGCATGTCAG-3’ |
| FGF2 | Forward  Reverse | 5’-GCGACCCACACGTCAAACTA-3’  5’-CCGTCCATCTTCCTTCATAGC-3’ |
| Fndc5 | Forward  Reverse | 5’-TCATTGTTGTGGTCCTCTTC-3’  5’-GCTCGTTGTCCTTGATGATA-3’ |
| IL-6 | Forward  Reverse | 5’-GTTCTCTGGGAAATCGTGGA-3’  5’-GGAAATTCGGGGTAGGAAGGA-3’ |
| Myostatin | Forward | 5’-CTGTAACCTTCCCAGGACCA-3’ |
|  | Reverse | 5’-TCTTTTGGGTGCGATAATCC-3’ |
| Follistatin | Forward | 5’-AGAGGAAATGTCTGCTTCCG-3’ |
|  | Reverse | 5’-CACCTCTCTTCAGTCTCCTG-3’ |
| Activin A (inhibin βA)  TGF-β | Forward  Reverse  Forward  Reverse | 5’-TGAAGAAGAGACCCGATGTCA-3’  5’-GCTTTCTGATCGCGTTGAG-3’  5’-GCAACAATTCCTGGCGTTACC-3’  5’-CGCTGAATCGAAAGCCCTGTA-3’ |
| 18S rRNA | Forward | 5’-CGGCTACCACATCCAAGGAA-3’ |
|  | Reverse | 5’-GCTGGAATTACCGCGGCT-3’ |

IGF-1, insulin-like growth factor-1; FGF2, fibroblast growth factor 2; Fndc5, fibronectin type III domain-containing 5; IL-6, interleukin-6; TGF-β, transforming growth factor-β.
