# Supplementary material for: Roles of Insulin-Like Growth Factor-1 in Muscle Wasting and Osteopenia in Mice with Hyponatremia
Source: Calcif Tissue Int. 2025 Apr 14;116(1):61. doi: 10.1007/s00223-025-01369-7 (PMC11996959; doi:10.1007/s00223-025-01369-7)
Supplement: Supplementary file 2 — Supplementary file2 (DOCX 18 KB) [file 223_2025_1369_MOESM2_ESM.docx]

**Table S2.** Relationships between IGF-1 mRNA levels in the gastrocnemius or soleus muscles and parameters of muscle or bone in mice.

|  | IGF-1 mRNA levels | | | | |
| --- | --- | --- | --- | --- | --- |
|  | Gastrocnemius muscle | |  | Soleus muscle | |
|  | r | *P* |  | r | *P* |
| **dDAVP**  BV/TV | 0.893 | <0.001 |  | 0.714 | 0.002 |
| CtBMD | 0.778 | <0.001 |  | 0.678 | 0.004 |
| Grip strength | 0.882 | <0.001 |  | 0.644 | 0.007 |
|  |  |  |  |  |  |
| **Furosemide**  BV/TV | 0.704 | 0.022 |  | 0.702 | 0.016 |
|  |  |  |  |  |  |
| CtBMD | 0.738 | 0.009 |  | 0.733 | 0.010 |
| Grip strength | 0.756 | 0.007 |  | 0.741 | 0.009 |

A simple regression analysis was performed on IGF-1mRNA levels in the gastrocnemius or soleus muscle, and trabecular BV/TV, cortical BMD, or grip strength 8 weeks after first injection of dDAVP or furosemide. GA, gastrocnemius; CtBMD, cortical bone mineral density.
