# Supplementary material for: Roles of Insulin-Like Growth Factor-1 in Muscle Wasting and Osteopenia in Mice with Hyponatremia
Source: Calcif Tissue Int. 2025 Apr 14;116(1):61. doi: 10.1007/s00223-025-01369-7 (PMC11996959; doi:10.1007/s00223-025-01369-7)
Supplement: Supplementary file 3 — Supplementary file3 (DOCX 17 KB) [file 223_2025_1369_MOESM3_ESM.docx]

**Table S3.** Relationships between serum IGF-1 levels and parameters of muscle and bone or IGF-1 mRNA levels in the gastrocnemius and soleus muscles of mice.

|  | Serum IGF-1 | |  | **Furosemide** | Serum IGF-1 | |
| --- | --- | --- | --- | --- | --- | --- |
| **dDAVP** | r | *P* |  |  | r | *P* |
| BV/TV | 0.761 | 0.001 |  |  | 0.715 | 0.013 |
| CtBMD | 0.782 | <0.001 |  |  | 0.684 | 0.020 |
| Grip strength | 0.844 | <0.001 |  |  | 0.725 | 0.012 |
| IGF-1 mRNA  GA muscle | 0.756 | 0.001 |  |  | 0.699 | 0.017 |
| Soleus muscle | 0.827 | <0.001 |  |  | 0.751 | 0.008 |

A simple regression analysis was performed on serum levels of IGF-1 and trabecular BV/TV, cortical BMD, or grip strength 8 weeks after first injection of dDAVP or furosemide. GA, gastrocnemius; CtBMD, cortical bone mineral density.
